# Racial Disparities in Diabetes Care and Outcomes for Patients with Visual Impairment: A Descriptive Analysis of the TriNetX Research Network

**DOI:** 10.21203/rs.3.rs-3901158/v1

**Published:** 2024-01-30

**Authors:** Charisse Madlock-Brown, Austen Lee, Jaime Seltzer, Anthony Solomonides, Nisha Mathews, Jimmy Phuong, Nicole Weiskopf, William G. Adams, Harold Lehmann, Juan Espinoza

## Abstract

**Background:** This research delves into the confluence of racial disparities and health inequities among individuals with disabilities, with a focus on those contending with both diabetes and visual impairment. **Methods:** Utilizing data from the TriNetX Research Network, which includes electronic medical records of roughly 115 million patients from 83 anonymous healthcare organizations, this study employs a directed acyclic graph (DAG) to pinpoint confounders and augment interpretation. We identified patients with visual impairments using ICD-10 codes, deliberately excluding diabetes-related ophthalmology complications. Our approach involved multiple race-stratified analyses, comparing co-morbidities like chronic pulmonary disease in visually impaired patients against their counterparts. We assessed healthcare access disparities by examining the frequency of annual visits, instances of two or more A1c measurements, and glomerular filtration rate (GFR) measurements. Additionally, we evaluated diabetes outcomes by comparing the risk ratio of uncontrolled diabetes (A1c > 9.0) and chronic kidney disease in patients with and without visual impairments. **Results:** The incidence of diabetes was substantially higher (nearly double) in individuals with visual impairments across White, Asian, and African American populations. Higher rates of chronic kidney disease were observed in visually impaired individuals, with a risk ratio of 1.79 for African American, 2.27 for White, and non-significant for the Asian group. A statistically significant difference in the risk ratio for uncontrolled diabetes was found only in the White cohort (0.843). White individuals without visual impairments were more likely to receive two A1c tests, a trend not significant in other racial groups. African Americans with visual impairments had a higher rate of glomerular filtration rate testing. However, White individuals with visual impairments were less likely to undergo GFR testing, indicating a disparity in kidney health monitoring. This pattern of disparity was not observed in the Asian cohort. **Conclusions:** This study uncovers pronounced disparities in diabetes incidence and management among individuals with visual impairments, particularly among White, Asian, and African American groups. Our DAG analysis illuminates the intricate interplay between SDoH, healthcare access, and frequency of crucial diabetes monitoring practices, highlighting visual impairment as both a medical and social issue.

## Background

Health disparities between people with disabilities and those without are poorly understood and under-researched. Factors that impact healthcare outcomes for disabled individuals include socioeconomic and environmental influences on healthcare system accessibility and quality of care.

In this paper, we argue that advanced causal modeling is necessary to help understand these factors and their interactions. Such an approach is crucial to improve data collection methods and ensure that real-world data effectively supports individuals with disabilities. Our study demonstrates disabilities like visual impairments intersect with race to influence the management of chronic conditions like diabetes. By examining disparities in the accessibility and quality of healthcare and subsequent health outcomes, this research illuminates the complex ways disability and race interact within healthcare systems. This understanding is vital in contributing to the broader discourse on health equity and developing informed healthcare strategies that effectively address these inequities.

Health disparities research has increasingly acknowledged the intersection of race, disability, and chronic disease management. In 2022, the National Institute for Minority Health and Health Disparity officially designated disabled populations as a vulnerable population. Approximately 67 million adults in the U.S. (27% of U.S. adults) are disabled. Adults with disabilities are more likely to be obese, smoke, have heart disease, and have diabetes: 41.6 % of adults with a disability are obese, while 29.6 percent of adults without a disability are obese; 21.9 % of adults with a disability smoke, while 10.9 percent of adults without a disability smoke; 9.6 % of adults with a disability have heart disease while 3.4 percent of adults without a disability have heart disease; 15.9 % of adults with a disability have diabetes while 7.6 percent of adults without a disability have diabetes.(1,2) In 2015, disability-associated healthcare expenditures accounted for 36% of all healthcare expenditures for adults residing in the United States, totaling $868 billion, with state expenditures ranging from $1.4 billion in Wyoming to $102.8 billion in California.(1,3,4) Healthcare spending for people with disabilities is determined by the cost of health-related services and the number of services used (1).

According to the Annual Report on People with Disabilities in America, the number of individuals with disabilities has continued to increase, from one in five in 2013 to one in four in 2016.(5) Disability-related disparities exist across several health-related measures (e.g., health-related behaviors, physical, mental, and oral health). (3,6,7). Addressing disability in the healthcare setting and its intersection with other forms of marginalization and minoritization is vital, but often overlooked, particularly in chronic conditions like diabetes.(5,8–10) This oversight leaves a significant portion of the population inadequately represented in health outcomes research. This lack of targeted research hinders the development of tailored healthcare strategies that address the needs of chronically ill populations. Existing studies may generalize findings across disabilities or fail to differentiate the impact of varying disabilities on health outcomes across racial groups. To bridge these gaps, we propose the following research questions:

1. Among patients with diabetes, are there significant differences in the prevalence of cardiopulmonary comorbidities between patients who are visually impaired versus those who are not?
2. Are there racial disparities in the quality of diabetes-related care between those who are visually impaired **not** due to diabetes-related ophthalmic complications versus those who are not visually impaired?
3. Are there racial disparities in health outcomes for persons with diabetes with a visual impairment diagnosis? How do these disparities compare to those with diabetes and no visual impairment diagnosis?

**Hypotheses:**

1. Patients with visual impairment will have higher HbA1c and a higher rate of chronic kidney disease regardless of race.
2. Patients with visual impairment will be less likely to have received the recommended standards of care for diabetes patients.
3. The disparities between those with visual impairment and those without these conditions, as identified in hypotheses 1 and 2, will be greater among racial minorities.

Additionally, we developed a directed acyclic graph (DAG) to represent possible causal pathways crucial to this proposal. DAGs are widely used in epidemiological research for their ability to visually represent known, assumed, or hypothesized relationships between variables, including risk factors, outcomes, and covariates.(11) DAGs can be used to guide study design and analyses and to identify potential biases.(12) They provide a structured framework to systematically evaluate and address these complexities, thereby enhancing the robustness and applicability of the research findings.

The impact of addressing these research questions is multifold. The study provides insights into how racial disparities and disabilities intersect to affect diabetes care and outcomes. This research will serve as a foundation for future studies, encouraging a more inclusive approach in health disparity research that accounts for the interplay of race and disability. Armed with this knowledge, disabled communities, particularly those managing chronic conditions like diabetes, can advocate more effectively for their healthcare. In addition, awareness of the impact of visual impairment and race as important factors in the quality of diabetes care can lead the way to improved systems of care in hospital systems and greater awareness for medical professionals.

## Methods

This study used a cross-sectional design analyzing real word electronic health record data. The study leverages data from the TriNetX Research Network, encompassing electronic medical records from approximately 115 million patients across 83 de-identified healthcare organizations.(13) The data used in this study was collected on November 29th, 2023. The study design is a cross-sectional analysis focusing on patients aged 45 and above who had healthcare visits between January 1, 2017, and December 31, 2018. This timeframe was chosen to avoid the confounding impact of the COVID-19 pandemic.

## Ethical Considerations

This TriNetX study is exempt from requiring informed consent because it is a retrospective study that involves secondary analysis of existing, de-identified data viewed only in the aggregate. This analysis does not involve direct intervention or interaction with human subjects. The de-identification of the data adheres to the standards defined in Section §164.514(a) of the HIPAA Privacy Rule.

### Terms used in this manuscript

We use person-first language throughout this manuscript, including people with diabetes and people with visual impairment.

### Classification of visual impairment

In the absence of either objective visual assessment data or self-reported visual disability status (neither of which is available in Trinetx), we used the presence of visual disability-related ICD-10 codes (here termed VDRC) to define four different categories of visual impairment: None, Unqualified, Low Vision, and Blindness. We identify people with visual impairment if they have a diagnosis code in the latter three categories. **Table 1** of the supplemental materials identifies the ICD-10 codes associated with each.

### Inclusion criteria

In our study, we aim to answer three distinct research questions, each with its own set of inclusion criteria for participant selection.

The first question investigated whether significant differences in baseline comorbidities are associated with disease burden between individuals with a VDRC and those without.

The criteria for inclusion in this analysis are individuals aged 45 years or older who had a healthcare visit between January 1, 2017, and December 31, 2018. This group is further divided into two cohorts: Cohort 1 comprises individuals without a VDRC (excluding those with diabetes-related ophthalmology complications in the baseline period), and Cohort 2 includes those with a VDRC (excluding those with ophthalmology complications in the baseline period).

The second research question explores if racial minorities are less likely than the White population to receive the standard of care recommended for diabetes patients, and how these trends vary by visual impairment status. Participants for this analysis are aged 45 or older, with a diabetes-related healthcare visit in the same period (January 1, 2017 to December 31, 2018). They must have had at least one visit post-2019, ensuring they were present throughout all of 2019 and able to have at least three ambulatory visits. This question is examined across three cohorts: Cohort 1, which includes individuals without a VDRC and with diabetes (excluding those with ophthalmic complications in the baseline period); and Cohort 2, comprising those without a VDRC (excluding those with diabetes-related ophthalmology complications).

The third question assesses whether racial minorities are more likely to have poorer diabetes outcomes compared to the White population among those receiving primary care. The inclusion criteria for this analysis are similar to the second question: participants must be 45 years or older, with a diabetes-related healthcare visit between January 1, 2017, and December 31, 2018, and at least one visit in 2019. The cohorts are identical to those in the second question, with Cohort 1 including those without a VDRC and diabetes not with ophthalmic complications, and Cohort 2 consisting of individuals with a VDRC and diabetes (excluding those with ophthalmic complications).

Through these criteria, the study aims to rigorously analyze the intersections of visual impairment status, racial disparities, and diabetes care and outcomes.

### Outcome variables

To explore disparities in disease burden, examine a selected set of comorbidities related to chronic pulmonary conditions identified by the Charlson Comorbidity Index (CCI).(14) We focused on this set of conditions as PwD have an increased risk of these diseases as well as an increased risk of mortality for asthma-associated hospitalization.(15) To explore whether racial minorities are less likely to receive standard diabetes care, the study measures outcomes such as having at least three ambulatory visits annually and receiving two or more A1C measurements within a year. Furthermore, to determine if racial minorities experience poorer diabetes outcomes, it identifies indicators like the prevalence of chronic kidney disease and the rate of uncontrolled diabetes (A1C > 9.0%) in the follow-up year. These outcome variables are crucial in understanding and addressing healthcare disparities among different racial groups.

### Healthcare Access Patterns

We examined several key aspects of healthcare utilization. For each of these outcomes, we identified the difference in proportion between patients with visual impairment and those without. First, we identified differences in the frequency of healthcare visits between patients with visual impairment and those without. Specifically, we identified whether patients had at least three ambulatory visits in the follow-up year. Next, we assess the frequency of diabetes monitoring. This part of the study specifically looked at whether patients had two A1c lab tests in the follow-up year. Lastly, we analyzed kidney disease monitoring, an important aspect of diabetes management. This was measured by the presence of at least one glomerular filtration rate (GFR) measurement. Each of these components contributes to a comprehensive understanding of healthcare access and utilization in the context of diabetes management.

### Statistical Analysis

#### Differences in Diabetes Outcomes Between White and Black Patients with Visual Impairment

The study examines the disparities in diabetes outcomes, particularly focusing on patients with visual impairment and comparing these outcomes between white and black patients. The analysis involves calculating the probability of Chronic Kidney Disease (CKD) in black patients with and without a VRDC by evaluating the risk of CKD in various strata based on VDRC and one-year follow-up using risk ratio.

#### Differences in Uncontrolled Diabetes (A1c>9.0%) in the Follow-Up Year

This study identified differences in the prevalence of uncontrolled diabetes between patients with visual impairment and those without these conditions using risk ratio. Uncontrolled diabetes was defined by a Hemoglobin A1c level greater than 9.0%, in the follow-up year.

#### Differences in Chronic Kidney Disease in the Follow-Up Year

This study identified differences in chronic kidney disease (CKD) prevalence between patients with visual impairment and those without these conditions using risk ratio. CKD was identified by the presence of any ICD-10 code starting with N18 (chronic kidney disease) in the follow-up year.

#### Comparing Co-morbidities Related to Chronic Pulmonary Disease

We used the differences in proportions test to understand how these comorbidities vary within the population.

### Directed Acyclic Graph (DAG) Inclusion

A key addition to our methods is the inclusion of a DAG to serve several purposes:

- **Identification of Confounders:** The DAGs helps differentiate covariates from potential confounders and identify transitive relationships impacting diabetes care and outcomes in patients with a
- **Exploring Missing Information:** It highlights challenges in using electronic health record (EHR) data for disability research, particularly how infrequently disability information is captured in EHR systems.
- **Articulations of Risk of Bias**: The DAG clarifies which variables are confounders but also which variables, if corrected for, induce bias.
- **Enhancing Study Interpretation:** By incorporating the perspectives of various stakeholders, the DAG ensures that the study results' interpretation comprehensively addresses the nuances of disability research within the context of EHR data.
- **Integrate research components**: The DAG motivates a range of research projects and articulates what research is needed, so the results cohere to provide a holistic understanding of the research problem.

The DAG in our study displays the pathways between having visual impairment and being measured as having uncontrolled diabetes.

### Results

Table 1 presents some significant findings regarding the prevalence of diabetes and its association with visual impairment among different racial cohorts. Notably, the incidence of diabetes was found to be nearly double in individuals with a VDRC across White, Asian, and African American groups. Within the cohorts having a VDRC Present, there was a lower proportion of female patients. Furthermore, the rate of ophthalmic complications varied among the cohorts: 23% in the African American group, 12% in the Asian group, and 17% in the White group.

Table 1
Demography and Diabetes Prevalence.

| Cohort | Race | Patient Count | Mean Age | % Female | % with Diabetes | % with Diabetes-Related Ophthalmic Complications |
|---|---|---|---|---|---|---|
| No VDRC | AA or Black | 1,524,072 | 64 | 61% | 31% | 4% |
| | White | 9,954,167 | 67 | 57% | 20% | 2% |
| | Asian | 456,630 | 65 | 61% | 23% | 3% |
| VDRC Present | AA or Black | 22,768 | 68 | 55% | 58% | 23% |
| | White | 105,287 | 72 | 54% | 46% | 12% |
| | Asian | 3,243 | 70 | 56% | 47% | 17% |

Table 2 focuses on the standard of care for diabetes patients among different racial cohorts receiving primary care, particularly in relation to visual impairment. It was observed that for the Asian, White, and African American cohorts, those with a VDRC showed a higher percentage of having three or more ambulatory visits annually, with a statistically significant p-value of less than 0.01. Additionally, visual impairment was linked to a higher risk of chronic kidney disease, with the White group having a higher risk ratio of 2.274. However, when it came to the risk ratio for uncontrolled diabetes, a statistically significant difference was only found in the White population, with a risk ratio of 0.843.

Table 2
Analysis of the proportion of patients with 3 + Ambulatory visits

| Race | (-) VDRC, % | (+) VDRC, % | Δ proportion (p-value) | CKD Risk Ratio (p-value) | Uncontrolled Diabetes Risk Ratio (p-value) |
|---|---|---|---|---|---|
| AA or Black | 70.33% | 81.15% | 10.82% (< 0.01) | 1.786 (< 0.01) | 0.968 (0.5) |
| White | 71.84% | 81.03% | 9.19% (< 0.01) | 2.274 (< 0.01) | 0.843 (< 0.01) |
| Asian | 63.50% | 75.22% | 11.72% (< 0.01) | 1.284 (0.08) | 0.847 (0.36) |

Table 3 in the study provides data on quality of care issues in diabetes management across different racial cohorts. Firstly, it was observed that in the White population, individuals without visual impairment were more likely to have two A1C measurements within the year 2019, indicating better diabetes management. However, this trend was non-significant in the Asian and African American cohorts. Secondly, across all populations, those with a VDRC present exhibited a higher risk of chronic kidney disease, with p-values being less than 0.02, suggesting a correlation between visual impairment and kidney disease in diabetic patients. Lastly, in contrast to the findings of Table 2, the risk ratio for having uncontrolled diabetes was only statistically significant in the White population, at 1.108.

Table 3
Analysis of the proportion of patients with 2 + A1C measures

| Race | (-) VDRC, % | (+) VDRC, % | Δ proportion (p-value) | CKD Risk Ratio (p-value) | Uncontrolled Diabetes Risk Ratio (p-value) |
|---|---|---|---|---|---|
| AA or Black | 27.66% | 28.09% | 0.43% (0.55) | 1.807 (< 0.01) | 1.106 (0.08) |
| White | 30.39% | 24.95% | 5.44% (< 0.01) | 2.3 (< 0.01) | 1.108 (0.01) |
| Asian | 32.42% | 28.90% | 3.52% (0.09) | 1.38 (0.02) | 0.909 (0.67) |

Table 4 of the study highlights significant differences in diabetes management across racial cohorts with at least one GFR measure, particularly in relation to visual impairment. In the African American population, those with a visual impairment code had a statistically higher rate of having a GFR measure compared to those without, suggesting more frequent kidney function monitoring in this group.

Conversely, in the White population, individuals with a visual impairment code were less likely to have a GFR measure, indicating a disparity in kidney monitoring for those with visual impairment. This trend was not observed in the Asian cohort, where the difference was not statistically significant. Additionally, the study found an increased risk of uncontrolled diabetes in those with a VDRC present significant in both White and African American populations, with the risk ratio being notably significant only for the White population at 0.9.

Table 4
Analysis of the proportion of patients with 1 + GFR measurements

| Race | (-) VDRC, % | (+) VDRC, % | Δ proportion (p-value) | CKD Risk Ratio (p-value) | Uncontrolled Diabetes Risk Ratio (p-value) |
|---|---|---|---|---|---|
| AA or Black | 60.20% | 64.82% | 4.62% (< 0.01) | 1.751 (< 0.01) | 0.979 (0.67) |
| White | 53.07% | 51.49% | 1.58% (< 0.01) | 2.203 (< 0.01) | 0.9 (< 0.01) |
| Asian | 57.41% | 58.53% | 1.12% (0.63) | 1.242 (0.04) | 0.989 (0.95) |

## Comorbidity analysis

Figures 1a to 1c in the study present a comparison of comorbidity burden within the chronic pulmonary domain, contrasting patients with visual impairment against those without. The findings reveal a significantly higher burden of comorbidities among cohorts with a VDRC present. Specifically, in the White cohort, the prevalence of asthma was 17% for those with a VDRC present, compared to only 10% for those without a VDRC present. Similarly, in the African American population, asthma rates were 20% for those with a VDRC present versus 13% for those without. In the Asian cohort, these figures were 14% and 9%, respectively. This pattern of higher comorbidity burden in patients with visual impairment was also consistent across other diagnoses.

The DAG developed in our study, shown in Fig. 2, offers comprehensive insights into the complex factors contributing to the detectability of uncontrolled diabetes among individuals with visual impairment, as recorded in the EHR. Patients with a disability are more than twice as likely to develop diabetes,(16), and that is reflected in our DAG, which puts Diabetes in the pathway between our primary risk factor and outcome. The DAG demonstrates the importance of understanding what causes patients to have diagnostic codes for disability in their medical records and how healthcare access and comorbid conditions are important drivers for including those codes. Recognizing that many individuals with visual impairment may not be adequately represented in these records is critical. The graph also emphasizes the impact of social drivers of health (SDoH) on healthcare access, impacting data completeness from healthcare visits to the measurement of A1C levels. This indicates that these values may be missing for patients without access to care. We could not adequately address this in our analysis due to the limitations of the TriNetX data. The DAG also depicts the relationship between healthcare site and documentation, as practices around documentation can vary significantly by site. The DAG informs our statistical analysis, with ‘Comorbid Condition’ being the sole variable identified in the minimal sufficient adjustment set for estimating the total effect of ‘Disability (Visual Impairment)’ on ‘Uncontrolled Diabetes.’ Controlling for comorbid conditions is essential to accurately estimate the total effect of visual impairment on uncontrolled diabetes. While we could not control all potential comorbid conditions that can cause visual impairment due to the capabilities of TriNetX, we removed patients with any code indicating they had diabetic ophthalmic complications for the patients with visual impairment cohort. Additionally, the DAG indicates that demographics are confounders in the relationship between diabetes and uncontrolled diabetes. While we could not adjust for all demographics, our race-stratified analysis allowed us to control for an important demographic variable. DAG code and details on the total and direct effects are presented in the supplemental materials.

## Discussion

The study assessed racial differences in co-morbidities, visit frequency, and diabetes control for people with visual impairment. Table 1 reveals that the incidence of diabetes is nearly double in individuals with a visual impairment code among White, Asian, and African American groups. The rate of ophthalmic complications also varies, being highest in the African American group. Table 2 highlights the standard of care in diabetes patients, showing that those with visual impairment in all racial cohorts had more ambulatory visits and a higher risk of chronic kidney disease, with significant disparities in uncontrolled diabetes risk ratios, particularly in the White population. Table 3 underscores quality-of-care issues, revealing that White individuals without a visual impairment code were more likely to have two A1c measurements within a year, a trend not observed in Asian and African American cohorts.

All populations with visual impairment codes showed a higher risk of chronic kidney disease. Among patients with at least two A1c readings, only the White population with a visual impairment code had a higher risk of uncontrolled diabetes. The study also examined comorbidity burdens in the chronic pulmonary domain, where Figs. 1a-1c demonstrated a higher burden among patients with visual impairment across all racial groups. The DAG analysis further elucidated the complex interplay of SDoH in diagnosing visual impairment and its consequential impact on diabetes management, highlighting the influence of SDoH on healthcare access and the frequency of A1C measurements. The DAG also pointed out that many people with visual impairment remain undetected in electronic health records. Collectively, these findings shed light on the intricate relationships between diabetes management, visual impairment, and racial disparities, emphasizing the need for tailored healthcare approaches in these diverse populations.

Including the DAG enhances our study interpretations as we identify important confounders (e.g., demographics, SDoH, and comorbid conditions) and other important variables in the causal pathway, such as healthcare sites. Identifying these variables is instrumental in understanding missing information and bias issues. The analysis from our study reveals the intersection between SDoH and diagnostic practices in influencing EHRs, specifically concerning visual impairment and diabetes management. This interaction potentially leads to Misclassification Bias and Surveillance Bias.(17) The DAG shows that disparities in healthcare access, shaped by SDoH, contribute to inconsistent documentation of visual impairment and diabetes-related metrics such as A1C measurements. Such disparities can result in Misclassification Bias, where groups with limited healthcare access may be underrepresented in EHRs regarding visual impairment or uncontrolled diabetes. Furthermore, the DAG indicates that individuals with visual impairment might receive more intensive health surveillance, particularly in A1C level measurement and monitoring for diabetes-related complications. This heightened monitoring could lead to Surveillance Bias, where the disparity in the frequency and depth of monitoring among different groups might distort the perceived prevalence or severity of diabetes and its complications. These biases highlight the critical need to consider the influence of social and healthcare access factors when interpreting EHR data. The study emphasizes the necessity of equitable healthcare practices to ensure accurate and inclusive representation in health research. Additionally, the DAG underscores the importance of accounting for variations in documentation practices across different sites to guarantee robust and reproducible results. Further, the DAG suggests the need for additional research focused on the variability in how visual impairment status is documented in EHRs, especially because the debiasing analyses suggested by the DAG requires site-identified, row-level data not available in the TriNetX network. This research should examine the consistency and accuracy of disability coding in EHRs, addressing issues like underreporting or misclassification. Such studies are crucial for improving patient management, epidemiological tracking, and informing policy, ensuring that EHR systems are more inclusive and effectively capture the needs of individuals with disabilities.

Our study's observation that individuals with visual impairment exhibit higher rates of chronic kidney disease across racial groups resonates with existing research on disability disparities. This finding is not surprising given that researchers have noted that the pathophysiology of kidneys and the eyes are linked.(18) Disability burden, regardless of the organ system involved, predicts worse outcomes and longer hospital stays during emergency medical admissions.(19) This aligns with our results, suggesting that visual impairment, as a form of disability, could be a marker for greater healthcare needs and more complex medical management, including the increased prevalence of chronic kidney disease.

Furthermore, Signore et al., (2021) reported risks associated with specific disabling conditions, such as those impacting pregnancy outcomes.(20) Their findings emphasize the need for tailored healthcare approaches, a perspective directly relevant to our study. Although our focus is on visual impairment and chronic kidney disease, the principles outlined by Signore et al. and the lack of systematic evidence in this area of healthcare quality underline the importance of our findings.(20) It suggests that, just as with pregnancy-related disabilities, visual impairment may require specialized care strategies to mitigate associated health risks like chronic kidney disease.

This pattern of increased comorbidity burden in people with visual impairment, particularly in the context of chronic pulmonary conditions like asthma, suggests several implications for healthcare practice and policy. Firstly, the findings indicate that visual impairment might be a marker for a greater overall health burden or reflect challenges in health management among those with visual impairments. This could be due to various factors, including but not limited to, difficulties in accessing healthcare services, challenges in self-managing chronic conditions, or a potential lack of tailored healthcare services for individuals with visual impairments. Moreover, the observed differences in comorbidity prevalence across racial groups suggest that underlying social determinants of health might influence these outcomes. Factors such as socioeconomic status, access to healthcare, and potential biases in healthcare provision could contribute to the disparities seen in comorbidity burdens. This is particularly relevant considering the role of demographics and social determinants of health as highlighted in our DAG analysis.

Our study's analysis of quality of care, as detailed in Tables 2 and 3, reveals a complex and sometimes inconsistent picture of healthcare utilization across different racial groups between people with visual impairment. From Table 2, we observed that individuals with visual impairment across all racial cohorts were more likely to have 3 + ambulatory visits annually compared to people without visual impairment, suggesting a higher engagement with healthcare services. Similarly, for the recommended A1c values, the White population with visual impairment were more likely to have A1c captured compared to those without visual impairment, and the results were non-statistically significant for the Asian and African American cohorts. As shown in Table 4, African Americans with visual impairment showed a higher frequency of kidney function tests compared to those without. In contrast, White individuals with similar visual impairments were less likely to undergo such tests, indicating a gap in their diabetes care. This trend was not statistically significant in the Asian population. Given these findings, it becomes evident that further analysis is essential to understand the nuances of diabetic care quality among people with visual impairment. This should include a deeper exploration of the factors influencing healthcare access and management strategies in these specific patient groups, thereby helping to identify and address gaps in care.

These findings align with and expand upon the research that found young adults with disabilities were more likely to visit emergency rooms and have a usual source of care when sick compared to those without disabilities.(21) However, this increased healthcare utilization did not necessarily translate into better healthcare outcomes. Verlenden et al. also reported that young adults with disabilities were more likely to delay medical care due to cost and had higher instances of unmet medical needs.(21) This parallel in our study suggests that while individuals with visual impairments (a form of disability) and diabetes may access healthcare services more frequently (as indicated by more ambulatory visits), this does not uniformly ensure adequate management of their condition (as shown by the inconsistent attainment of recommended A1C values).

Our findings highlight the complexities of healthcare access among racially diverse populations with disabilities, demonstrating that increased healthcare interactions do not always equate to improved healthcare outcomes. This underscores the need for a more nuanced understanding of healthcare utilization patterns among people with disabilities and tailored interventions to ensure equitable and effective healthcare delivery.

## Limitations

Our study, while providing valuable insights into diabetes management and visual impairment across racial cohorts, is subject to certain limitations. One significant limitation stems from the use of TriNetX, a healthcare data analytics network. While TriNetX offers a robust platform for data analysis, it does not allow for the inclusion of confounding variables, which could influence the study's outcomes as our DAG indicated variables such as healthcare site and SDoH play pivotal roles in the causal pathway between visual impairment and uncontrolled diabetes. This limitation restricts our ability to fully account for all factors that may affect the results, possibly leading to residual confounding. Furthermore, our reliance on ICD-10 codes to identify people with disabilities presents another challenge. Although efforts have been made to use ICD codes to capture disability, these are hindered by a lack of codes that adequately reflect functional status. The accuracy and comprehensiveness of data obtained from ICD codes are often compromised by financial and administrative incentives that influence diagnosis coding. This can lead to a limited scope and high variability in results, underlining the insufficiency of current methods in accurately capturing and addressing disability-related health disparities. Lastly, our study highlights the necessity for incorporating social model data collection to improve these results. The focus should not be limited to patients with a medical reason for having a visual impairment diagnosis in their records. Understanding disparities in healthcare requires a more holistic approach that includes social determinants of health. Such an approach would provide a more comprehensive understanding of the barriers faced by individuals with disabilities in accessing and receiving appropriate healthcare. Therefore, future research should aim to integrate social model data collection, which could provide deeper insights into healthcare disparities and guide more effective interventions for people with disabilities.

## Conclusion

This study addresses racial differences in gaps in the impact of visual impairment on health outcomes in diabetic patients across different racial groups. Our findings reveal significant disparities, with the incidence of diabetes nearly doubling in individuals with visual impairments across White, Asian, and African American groups. People with visual impairment experienced more healthcare visits and a higher risk of chronic conditions like kidney disease. Notably, White people without visual impairments were more likely to receive adequate diabetes monitoring, a trend absent in other racial cohorts.

The DAG analysis in our study highlighted the complex role of SDoH in visual impairment diagnosis and its impact on diabetes management. It pointed to the influence of SDoH on healthcare access and frequency of A1C measurements, suggesting that visual impairment is both a medical and social issue. This research underscores the need for nuanced healthcare approaches considering racial disparities and social factors. It calls for future studies to explore these intersections further, aiming to improve healthcare outcomes for diabetic people with disabilities across diverse racial backgrounds.

## Abbreviations

Directed acyclic graph (DAG)

Social drivers of health (SDoH)

## Declarations

## Ethics approval and consent to participate

This TriNetX study is exempt from requiring informed consent because it is a retrospective study that involves secondary analysis of existing, de-identified data viewed only in the aggregate. This analysis does not involve direct intervention or interaction with human subjects. The de-identification of the data adheres to the standards defined in Section §164.514(a) of the HIPAA Privacy Rule.

## Availability of data and materials

The data supporting this study's findings are available from the TriNetX Analytics Network. https://trinetx.com.

## Competing Interests

JE is a paid consultant for Sanofi. Sanofi had no role in this work.

## Consent for publication

Not applicable.

## Funding

This research received no external funding. This work was partially supported by the National Institutes of Health (NIH/NCATS U24TR002306 and U24TR002306-04S3 to JP; NIH/NCATS UL1TR001422 to JE). HL was supported by NCATS UL1TR003098 - 131418. NW was supported by the NLM (R01LM013990). WA was supported by the NCATS (1UL1TR001430). AS was supported by NCATS (UL1TR002389: subcontract to NorthShore), and the Research Institute, NorthShore University HealthSystem. The content is solely the responsibility of the authors and does not necessarily represent the official views of the National Institutes of Health. CMB was supported by salary support from the University of Iowa College of Nursing.

## Authors' contributions

Conceptualization, C.M.-B., J.E.; methodology, C.M.-B., J.E., W.G.A, H.L.; formal analysis, C.M.-B., A.L.; writing–original draft preparation, C.M.-B., J.S., A.S.; writing– review and editing, C.M.-B., J.E., W.G.A, H.L., N.M., J. P., J.S., A.S., A.L., N.W.; supervision, C.M.-B., J.E. All authors have read and agreed to the published version of the manuscript.

## Acknowledgments

We thank the University of Iowa Institute for Clinical and Translational Science for assistance with using the TriNetX system.

## Figures

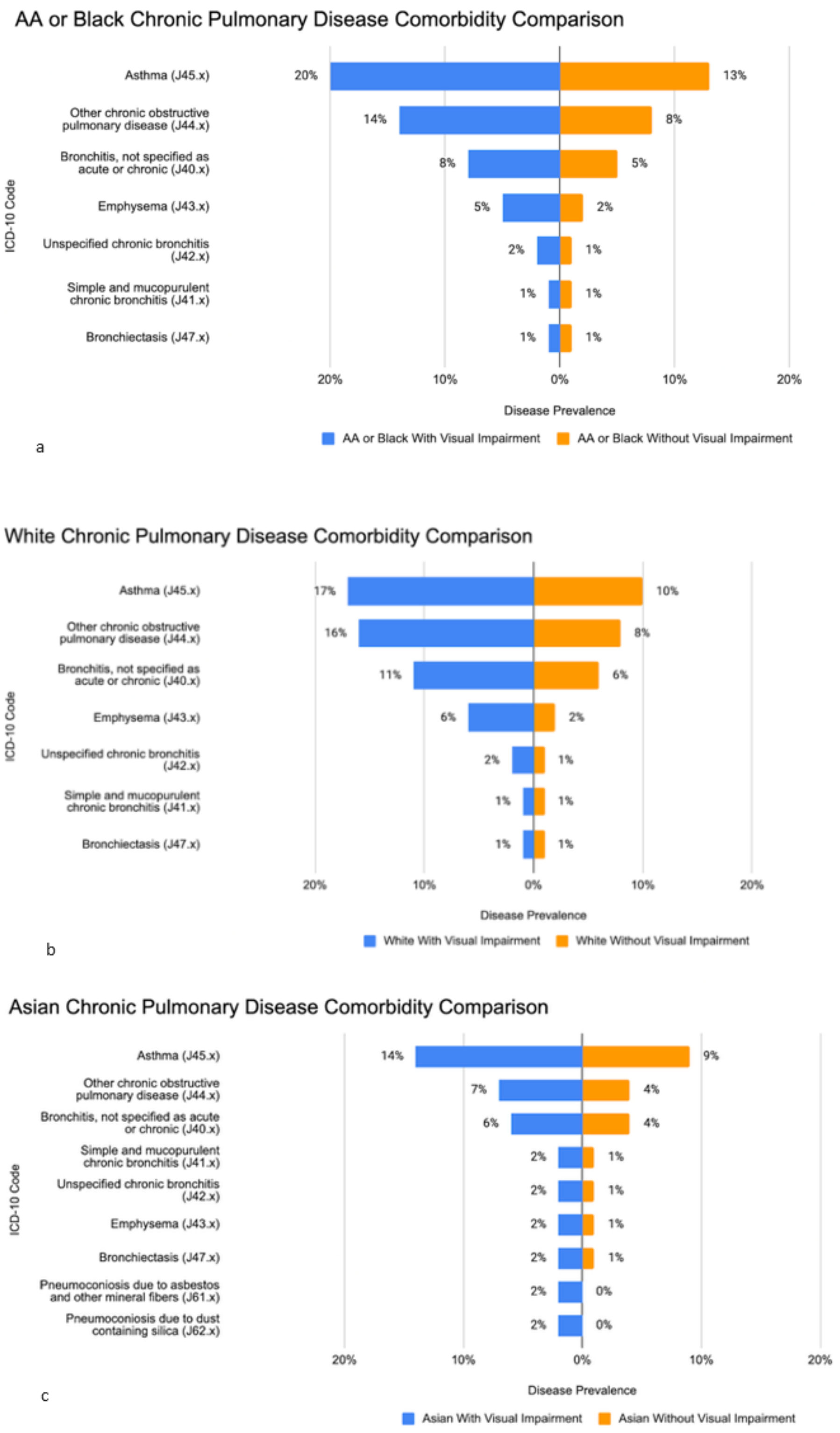

### Figure 1

**a.** AA or Black Chronic Pulmonary Disease Prevalence Comparison

**b.** White Chronic Pulmonary Disease Prevalence Comparison

**c.** Asian Chronic Pulmonary Disease Prevalence Comparison

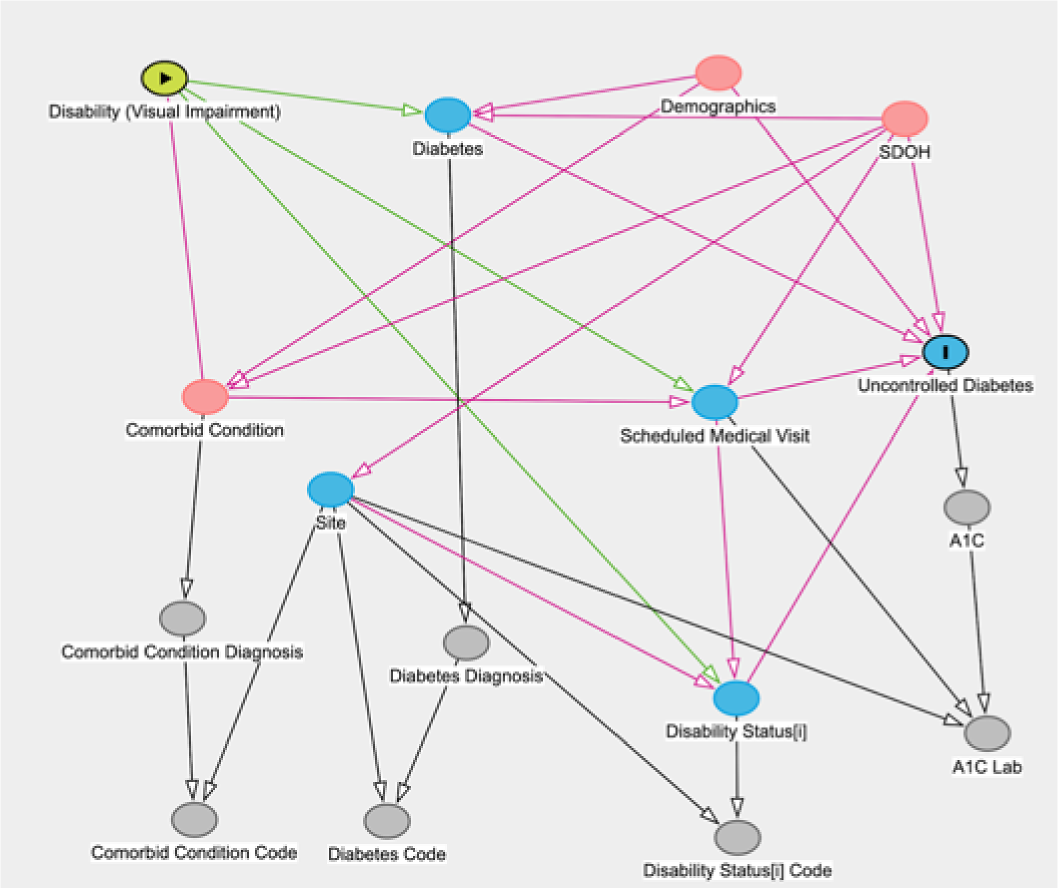

**Figure 2**

The DAG features an arrow from 'Disability (Visual Impairment)' to 'Uncontrolled Diabetes,' illustrating the hypothesized direct causal effect.

## Supplementary Files

This is a list of supplementary files associated with this preprint. Click to download.

- SupplementalMaterials.docx
